# Particle Fabrication Using Inkjet Printing onto Hydrophobic Surfaces for Optimization and Calibration of Trace Contraband Detection Sensors

**DOI:** 10.3390/s151129618

**Published:** 2015-11-24

**Authors:** Greg Gillen, Marcela Najarro, Scott Wight, Marlon Walker, Jennifer Verkouteren, Eric Windsor, Tim Barr, Matthew Staymates, Aaron Urbas

**Affiliations:** National Institute of Standards and Technology, Materials Measurement Laboratory, 100 Bureau Drive, Gaithersburg, MD 20899, USA; E-Mails: marcela.najarro@nist.gov (M.N.); Scott.wight@nist.gov (S.W.); Marlon.walker@nist.gov (M.W.); Jennifer.verkouteren@nist.gov (J.V.); eric.windsor@nist.gov (E.W.); timothy.barr@nist.gov (T.B.); matthew.staymates@nist.gov (M.S.); aaron.urbas@nist.gov (A.U.)

**Keywords:** inkjet printing, drop-on-demand, particle fabrication, sample preparation, hydrophobic silicon, trace detection

## Abstract

A method has been developed to fabricate patterned arrays of micrometer-sized monodisperse solid particles of ammonium nitrate on hydrophobic silicon surfaces using inkjet printing. The method relies on dispensing one or more microdrops of a concentrated aqueous ammonium nitrate solution from a drop-on-demand (DOD) inkjet printer at specific locations on a silicon substrate rendered hydrophobic by a perfluorodecytrichlorosilane monolayer coating. The deposited liquid droplets form into the shape of a spherical shaped cap; during the evaporation process, a deposited liquid droplet maintains this geometry until it forms a solid micrometer sized particle. Arrays of solid particles are obtained by sequential translation of the printer stage. The use of DOD inkjet printing for fabrication of discrete particle arrays allows for precise control of particle characteristics (mass, diameter and height), as well as the particle number and spatial distribution on the substrate. The final mass of an individual particle is precisely determined by using gravimetric measurement of the average mass of solution ejected per microdrop. The primary application of this method is fabrication of test materials for the evaluation of spatially-resolved optical and mass spectrometry based sensors used for detecting particle residues of contraband materials, such as explosives or narcotics.

## 1. Introduction

Trace contraband detection refers to the use of chemical sensors designed to screen for the presence of particle residues that may be found on surfaces as a result of direct or secondary contact with an explosive or illicit narcotic. Most current implementations of this widely-deployed technology involve the collection of micrometer-sized particles from a suspect surface using a sampling swipe. The swipe is subsequently heated to produce contraband vapor signatures, which are then analyzed by ion mobility spectrometry (IMS) [1]. Assuming adequate particle collection efficiency on the sampling swipe, the individual particle characteristics of the residues are not critical for a successful IMS detection as the analytical response of these sensors is primarily dependent on the total mass of analyte collected on the swipe. However, many emerging analysis techniques for trace contraband detection, including both optical [2] and mass spectrometric techniques [3,4,5,6], provide direct spatially-resolved chemical analysis of individual residue particles, making the spatial distribution, surface coverage, and the size and shape of contraband particles on the surface critically important considerations for effective detection. While there is a lack of information on the particle characteristics of contraband residues in “real world” scenarios, a number of laboratory-based studies [7,8,9,10,11,12] provide some broad generalizations that suggest particle diameters ranging from submicrometer to hundreds of micrometers with “typical” particle sizes in the 10–20 micrometer diameter range and total masses of the deposit from a few nanograms to tens of micrograms. Areal surface coverage (fill factor) of contraband particles in fingerprint residues are typically in the sub-1% range [10].

In order to appropriately evaluate the performance of direct analysis trace contraband sensor technologies, there is a need for realistic particle test materials that provide a high degree of control over total concentration, particle size, shape, and surface coverage. There are various methods for preparation of such materials, several of which are summarized in a recent review of standards for optically-based trace detection technologies [13]. The most widely used sample preparation method is drop casting from solution, where a known volume of solution is dispensed, typically by pipette, onto a surface and allowed to dry, forming a solid deposit. This approach often leads to the formation of a “coffee ring” deposit [14] with higher mass loadings on the droplet periphery and variable particle morphology across the deposit. Drop casting does not provide a high level of control over the physical characteristics of the sample, and is influenced by the characteristics of the solvent, analyte concentration, and wetting characteristics of the surface.

More recently, inkjet printing, using “inks” containing explosives or narcotics dissolved in an appropriate solvent, has been gaining increased attention as a method to prepare samples for validating and optimizing the response of various trace chemical sensors to a known mass of contraband material [15,16,17,18,19,20,21,22,23,24]. The current study builds on earlier efforts to develop inkjet printing for quantitative solution deposition [17,24,25,26] while adding critical control and characterization of additional factors in the printed deposits. These factors include the particle shape (width and height) while maintaining the ability to produce particles in arrays with specified density to control the fill factor. We demonstrate this approach using ammonium nitrate (AN), due to ease of handling and inclusion as a principal component of ammonium nitrate fuel oil (ANFO) explosives. An additional new development is a substrate treatment that provides high surface contact angles for a variety of surfaces and solvents and that allows for constrained inkjet droplet evaporation in a constant contact angle mode. We demonstrate that by using concentrated ink solutions and by controlling the number of microdrops/spot, the contact angle of the substrate (through the surface treatment), and the spacing of the printed arrays, we are able to provide tunable particle arrays with precise control over the mass per particle, particle height, particle diameter, and fill factor. The ability to “print particles” in predefined locations with well-known characteristics is necessary for more robust evaluation of sensors systems in which the analytical response may be critically dependent on particle size and morphology of the sample. We view the current work as an important contribution to the continued evolution of inkjet-based standards development for trace contraband detection.

## 2. Experimental Section

### 2.1. Gravimetric Preparation of Stock Ammonium Nitrate Solution

Research-grade ammonium nitrate (AN) (99%) was purchased from J.T. Baker (Phillipsburg, NJ*, USA). For these experiments, 250 mg of AN was dried to constant weight at 115 °C and was dissolved in a calibrated volume of deionized water obtained from Millipore Synergy Ultrapure Water System equipped with a 0.22 micrometer filter (Billerica, MA, USA). Ultrasonication was used to aid dissolution. The final gravimetrically-derived concentration was (22.973 ± 0.045) μg/mL with uncertainty reported as the standard deviation of multiple mass measurements. The concentration of the solution was validated by independent concentration measurement using UV-VIS spectroscopy as described briefly below.

### 2.2. Sample Substrate Preparation

Fluorinated silane monolayers were prepared on cleaned, one-inch diameter silicon wafers (Virginia Semiconductor, Fredericksburg, VA, USA). The wafers were exposed to UV light in a UV ozone cleaner for 15 min. to remove adventitious hydrocarbons and promote the formation of an oxide layer. The samples were rinsed with isopropyl alcohol and placed into a desiccator until ready to use. The wafers were exposed to perfluorodecyltrichlorosilane (FDTS) vapor in another desiccator for at least 24 h to facilitate the formation of a fluorinated hydrophobic monolayer. Typical water contact angles of wafers prepared in this manner are ≈106°. The water contact angle of the treated surface can be modified by exposure to UV light (in a UV ozone cleaner, for instance) to generate surfaces with water contact angles varying from ≈106° to less than 20°.

### 2.3. Inkjet Printing

Particle array samples were produced on the silane-treated silicon wafers using a Jetlab 4xLB gravimeter inkjet printer system (MicroFab, Plano, TX, USA). The Jetlab 4xLB is a drop-on-demand inkjet printing system with precision X, Y, Z motion control, drop ejection drive electronics, integrated pressure control, mass balance for drop mass measurement, and a drop visualization system. The dispenser consists of a glass capillary tube with a nominal 50 μm diameter orifice surrounded by a piezoelectric crystal. A trapezoidal voltage pulse (30 volts amplitude; rise time = 3 μs; dwell time = 30 μs; fall time = 3 μs) was applied to the piezo crystal to produce an acoustic wave that propagates through the aqueous ammonium nitrate solution in the capillary tube. When an acoustic wave of sufficient energy reaches the orifice, a microdroplet is ejected. For stable droplet ejection, the dispenser conditions were tuned by visual observation of the ejected microdrops, using stroboscopic illumination, while adjusting the voltage pulse parameters and the backfill pressure on the fluid in the capillary. Printing was performed at a frequency of 300 Hz with a typical droplet ejection velocity of ≈2 m/s and typical drop volumes of ≈50 pL. For printing of non-aqueous solvents (see conclusions), such as acetonitrile, a 20 micrometer diameter orifice inkjet dispenser was used.

Measurements of the drop mass were made using a Sartorius SE Series micro balance (Gottingen, Germany) using procedures described in detail elsewhere [26]. Briefly, after the drop ejection was stable, as determined by visual observation, the inkjet dispenser was placed over a specially configured weighing boat on the microbalance. The boat was first prefilled with solution to saturate the environment and establish a constant evaporation rate. Subsequently, a known number of drops were ejected and the mass/microdrop was calculated by dividing the total dispensed mass (corrected for evaporation) by the number of ejected microdrops. Measurements were made until repeat mass values agreed to within less than 1%.

### 2.4. Ultraviolet/Visible Absorption Spectroscopy (UV-VIS)

UV-VIS absorption spectroscopy measurements were used to verify the starting solution concentration and were performed using a Shimadzu UV-1800 (Kyoto, Japan) double beam spectrophotometer operated in photometric mode. Five point calibration curves were made by taking aliquots from an explosive standard solution (0.1 M Dionex, Thermo Scientific, Waltham, MA, USA) and diluting to concentrations that spanned the linear dynamic range for absorbance. Absorbance was measured using matched quartz cuvettes with a light path of 10 mm. One cuvette was filled with 1 mL of the explosive standard solution; the other functioned as a blank and was filled with water. Absorbance was then measured using wavelength scans from 196 nm to 275 nm.

### 2.5. Environmental Scanning Electron Microscopy

The scanning electron microscope (SEM) used in this work was an FEI Company (Hillsboro, OR, USA) Quanta 200F environmental scanning electron microscope (ESEM). For the results reported here, the samples were analyzed in high vacuum mode with electron beam energies of 1.5 keV to 10 keV. For particle diameter measurements, calibration of the image magnification was performed by the manufacturer to less than 3.0% error with a magnification reference standard (Geller Microanalytical, Topsfield, MA, USA). For particle height measurements, the stage was tilted to 89°, and no correction was applied for the foreshortening as it would have a negligible effect under these conditions. All measurement errors for height and diameter are reported as standard deviation.

### 2.6. Raman Spectroscopy

Raman Spectroscopy was conducted on individual ammonium nitrate particles using a Renishaw S1000 micro-Raman system (Renishaw, Gloucestershire, UK) consisting of a Leica DMLM microscope coupled to a 250 mm focal length imaging spectrograph with a proprietary deep depletion, thermoelectrically cooled (70 °C) charge-coupled device. For this work, a 532.2 nm continuous wave laser (Model 142, Lightwave Electronics, Mountain View, CA, USA), holographically ruled 1800 grooves/mm grating, and 5× to 50× objectives were used. Laser power at the sample was typically 1 mW to 10 mW and acquisition times of 1 s to 10 s were used. The Raman shift axis was calibrated using established peak positions of neat benzonitrile [27].

## 3. Results and Discussion

**Figure 1:**
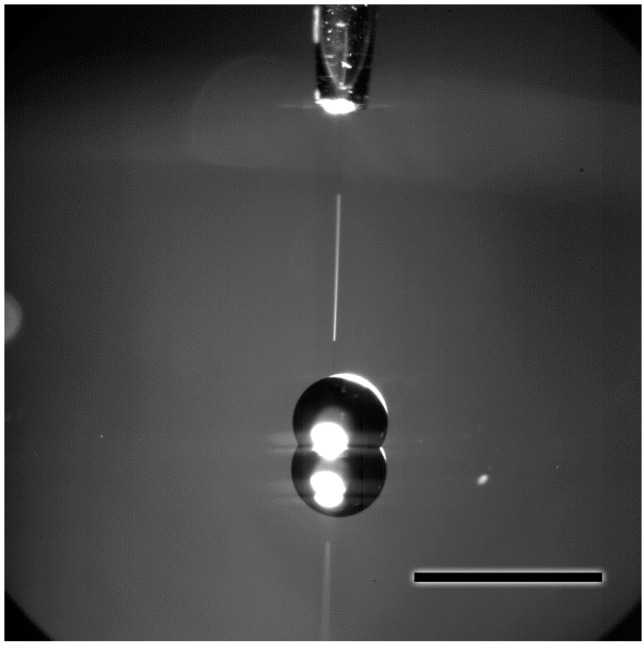
Image showing an optical microscope image of a single inkjet droplet (trail) being ejected from the inkjet printer head (top) onto the FTDS treated silicon substrate. The larger ensemble droplet on the surface contains several thousand individual drops. Note that on the reflective silicon surface we can see the mirror image of the drop. Scale bar in the image is 2 mm.

**Figure 2:**
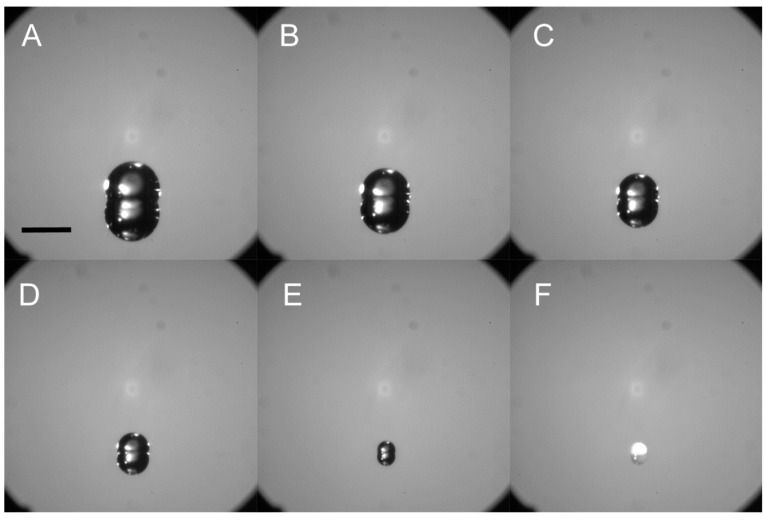
A series of time lapse optical microscope images (**A**–**F**) of several thousand microdrops of an ammonium nitrate solution inkjet-printed onto the silane coated silicon. The collection of multiple microdrops forms a larger ensemble drop that maintains a spherical cap geometry and rapidly evaporates leaving a solid residue. Each image in the series was acquired 18.5 s apart. The scale bar is 4 mm.

Figure 1 illustrates a typical particle fabrication experiment. The inkjet printer was used to dispense one or more microdrops of the concentrated ammonium nitrate solution onto the treated silicon wafer substrate with water contact angles of ≈106°. Unlike an inkjet dispensing onto a wetting substrate (untreated silicon, for example) the microdrops on smooth hydrophobic surfaces take on a spherical cap shape that is maintained during evaporation. This effect is illustrated in Figure 2 which shows a series of time lapse photos of the evaporation process ending with solidification/crystallization and formation of a particle deposit. The size of each solid deposit is a function of the total amount of analyte, which is controlled by the solution concentration and the volume deposited. In inkjet printing, the fundamental unit of solution delivery is a single microdrop, which, in this case, has a volume of approximately 50 pL. Any number of microdrops from 1 to 999 can be ejected in a single burst at the selected printing frequency to form an ensemble drop. To dispense more than 999 droplets at one location, more than one burst must be dispensed (this is a limitation imposed by our particular inkjet system). In this manner, a wide range of dispensed volumes can be achieved by simply varying the number of microdroplets. The examples shown in Figure 1 and Figure 2 show the extreme case with volumes approaching those dispensed by pipette. This was done for illustration purposes, and more typically, we work with less than 1000 microdrops per spot.

The evaporation behavior of the ensemble drop as determined from the time lapse imaging shown in Figure 2 appears to be consistent with the constant contact angle mode, with the droplet contact radius continually decreasing as evaporation continues. These observations are qualitative as detailed contact angle measurement could not be performed under these conditions. This behavior has been observed for sessile water droplets on smooth Teflon-coated surfaces [28]. The evaporative behavior occurs for ensemble drop volumes from μL size (those seen in Figure 2) down to single microdrops. Therefore, given a constant solution concentration and surface characteristics, the deposit size is selected by choosing the total volume of the ensemble drop. Using this approach, we have been able to fabricate solid ammonium nitrate particles ranging in size from a few micrometers to several hundred micrometers in diameter.

The procedure is amenable to other substrates that are smooth and are intrinsically, or can be rendered, hydrophobic. Substrates evaluated to date include Teflon, aluminum, copper, glass, TiO_2_, and several cloth substrates made hydrophobic by plasma modification. Superhydrophobic surfaces (contact angles >150°) prepared on silicon were also examined as potential substrates for this application, but the inkjet drops exhibited a propensity to either bounce or roll off of this surface making fabrication of arrays on discrete locations problematic.

**Figure 3:**
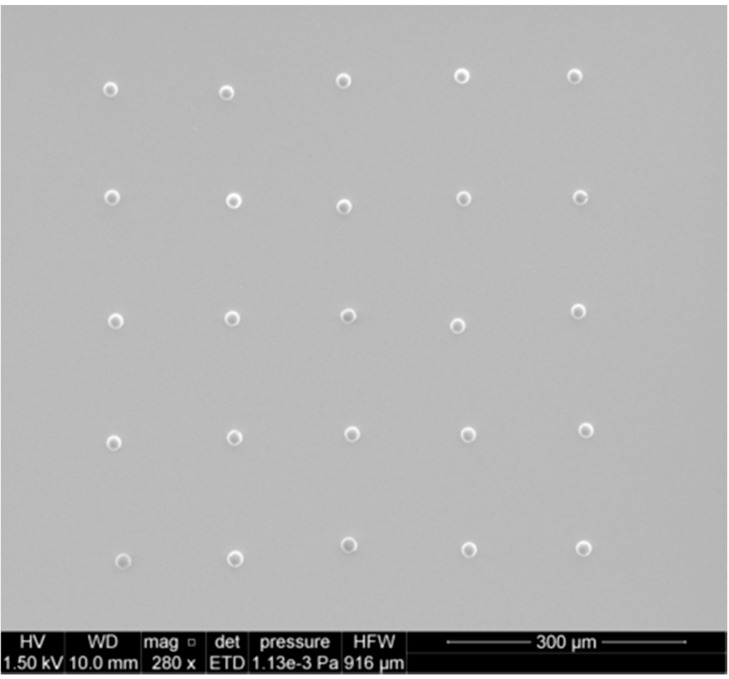
5 × 5 particle array of ≈18 μm diameter AN particles printed onto hydrophobic functionalized silicon substrate with a nominal spacing of 150 μm between particles. Each particle was formed by evaporation of an ensemble drop of 5 inkjet microdrops containing ammonium nitrate deposited onto the same sample location.

With the ability to select particle (deposit) size, the next step in sample fabrication is the ability to deposit multiple particles. Arrays of particles can be printed by sequential translation of the sample stage, which is easily attained through the scripting language available with inkjet printers. Figure 3 shows an array of ≈18 μm diameter ammonium nitrate particles fabricated on the silane-treated silicon. The spacing between particles, in this example 150 μm, is adjusted by modification of the stage translation distance. The spreading of ensemble drops will limit the minimum spacing, unless overlap of ensemble drops is acceptable. In general, we prefer to select array spacings to avoid overlap and, thereby, maintain control over particle size afforded by volume selection.

**Figure 4:**
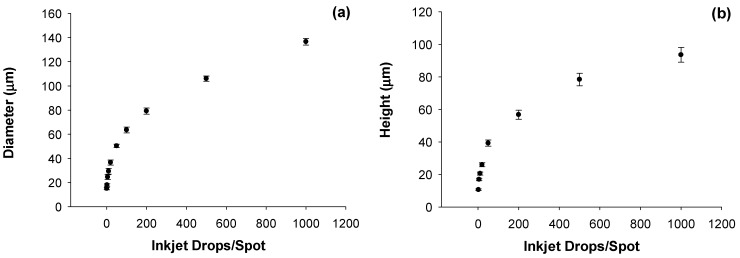
(**a**) Individual particle diameters as measured from a series of arrays of increasing numbers of inkjet microdrops/spot using scanning electron microscopy; (**b**) Measured particle heights measured from a series of arrays of increasing particle height as measured using scanning electron microscopy at oblique incidence angles. Error bars represent the standard deviation of multiple measurements (n) with n varying from 3 to 16.

In order to calibrate the diameter and size of the individual dried particles we use both normal and oblique incidence environmental scanning electron microscopy (ESEM). This is demonstrated in Figure 4a,b for 4 × 4 particle arrays fabricated by dispensing the standard ammonium nitrate solution in water. Ten different arrays were formed by varying the number of microdrops in each spot from 1 to 999. The particle arrays comprise solid spherical capped-shaped particles with diameters increasing from 15 μm ± 1.1 μm (1 microdrop/spot) to 136 μm ± 2.8 μm (999 microdrops/spot) with corresponding particle heights increasing from 10.62 μm ± 0.7 μm to 93.52 μm ± 7.3 μm. Uncertainties in particle size and shape from the ESEM data are reported as one standard deviation for multiple (n) measurements with *n* = 3 to *n* = 16. The individual particle masses vary from 1.2 ng for one microdrop/spot to 1.23 μg for 999 microdrops/spot. Of note for this data set is the observed repeatability of the microdrop mass measurement. Before the array samples were prepared, the gravimetric determination of inkjet microdrop mass was 53.55 ng or 53.55 pL ± 0.142 pL (*n* = 6, 0.36% RSD). After one hour of printing arrays, the measured mass was 53.10 ng or 53.10 pL ± 0.122 pL (*n* = 4, 0.23% RSD) representing a drift in drop volume of less than 1%.

The high degree of precision of the inkjet printing allows for fabrication of particle arrays that are highly controllable in terms of individual particle characteristics as well as the spatial distribution of these particles on a surface. This degree of control over fabrication of test materials is a critical requirement for eventual evaluation of both large-area sensors that are sensitive to the area coverage of the particles on the surface, as well as for sensors that have sufficient spatial resolution to interrogate individual particles.

**Figure 5:**
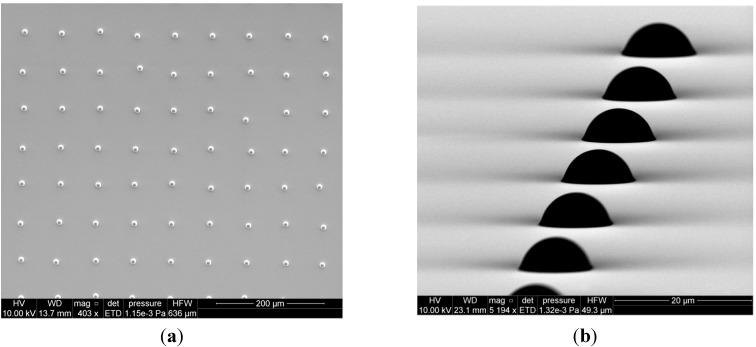
(**a**) Printed array of inorganic salt particles containing multiple elements in 3% nitric acid solution. The standard solution contains Ca, Mg, K, Li, Na, Cr, B, Fe, Mn, K, Au, Zn, Al, Cu, Ni, ^6^Li, ^204^Pb, ^25^Mg, ^54^Fe, ^50^Cr, ^10^B at concentrations ranging from 50 to 1000 μg/mL; (**b**) High tilt scanning electron micrograph of several particles from the array.

Using the approach described above, a variety of particle array sensor test samples have been fabricated from water soluble analytes. This includes inorganic explosive materials, such as potassium chlorate, pharmaceuticals, illicit narcotics, and trace metals. Test materials containing trace metals samples are also relevant to contraband detection sensors as metals are often used as additives in various improvised explosive devices. Test materials would be needed for chemical sensing techniques, such as X ray fluorescence, laser induced breakdown spectroscopy, or secondary ion mass spectrometry, that respond to the presence of elemental species. Figure 5a shows a portion of a 10 × 10 particle array with 50 μm spacing between particles produced by printing a commercially available trace element dilute nitric acid solution standard (Inorganic Ventures, Christiansburg, VA, USA). This standard solution contains the elements: Ca, Mg, K, Li, Na, Cr, B, Fe, Mn, K, Au, Zn, Al, Cu, Ni, ^6^Li, ^204^Pb, ^25^Mg, ^54^Fe, ^50^Cr, ^10^B at concentrations ranging from 50 to 1000 μg/mL. Figure 5b shows a high tilt SEM image of the sample demonstrating the reproducibility of the individual particle deposits.

## 4. Chemical Analysis of Printable Arrays

**Figure 6:**
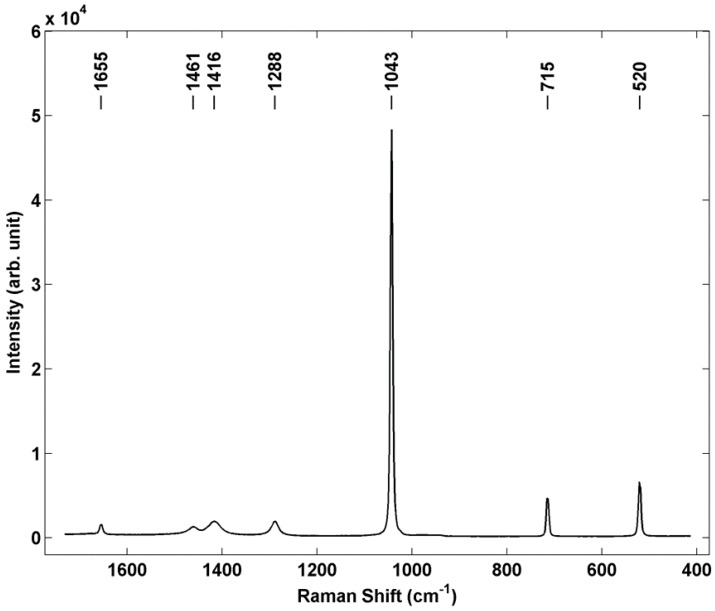
Micro Raman spectrum of individual AN particle prepared by inkjet printing. Renishaw S1000 Micro Raman system, 532 nm excitation, 50×, ≈1 mW power at sample, 10 s integration.

Raman microspectroscopy was used as a representative chemical sensing technique to demonstrate the potential applicability of using individual particle array samples for performance optimization and characterization of non-contact sensors. In these experiments a Renishaw S1000 micro Raman system was used at 532 nm excitation, and a spectrum from a single printed AN particle is shown in Figure 6. This particular AN particle has a deposited mass of 6.15 ng, a diameter of 24.5 μm and height of 16.9 μm. The spectrum shows representative AN bands at 716 cm^−1^ and 1043 cm^−1^ [29], as well as the silicon band at 520 cm^−1^. Band assignments for AN are shown in Table 1. Ammonium nitrate exhibits significant polymorphism, with five known phases existing up to the melting temperature under ambient pressure conditions [30,31]. It has also recently been found that inkjet printing of AN can lead to different polymorphs depending on both the concentration of the starting ink solution as well as the nature of the substrate on which it is printed [32]. Based on identification of the nitrate stretch at 1043 cm^−1^ and the printing conditions used, we infer that the orthorhombic phase four, which is the room temperature phase, is dominant in these printed samples.

**Table 1:** Raman band assignments for ammonium nitrate.

Raman Shift (cm^−^^1^)	Band Assignment
715	$\nu_{4}$ (NO_3_^−^), (A_g_, B_1g_)
1043	$\nu_{1}$ (NO_3_^−^), A_g_
1288	$\nu_{3}$ (NO_3_^−^), A_g_
1416	$\nu_{3}$(NO_3_^−^), B_1g_
1461	$\nu_{4}^{\prime }$(NH_4_^+^), B_1g_
1655	$2\nu_{2}$ (NO_3_^−^), A_g_

**Figure 7:**
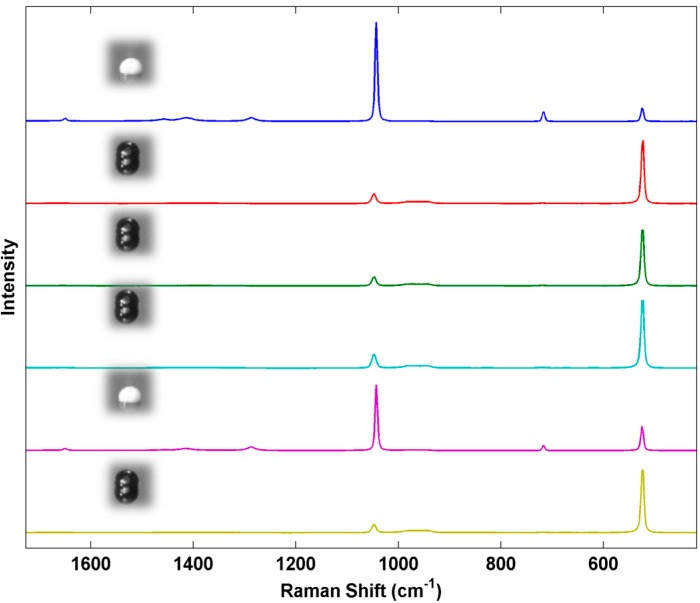
Comparison of Raman spectra of inkjet test materials containing both liquid and solid AN deposits.

**Figure 8:**
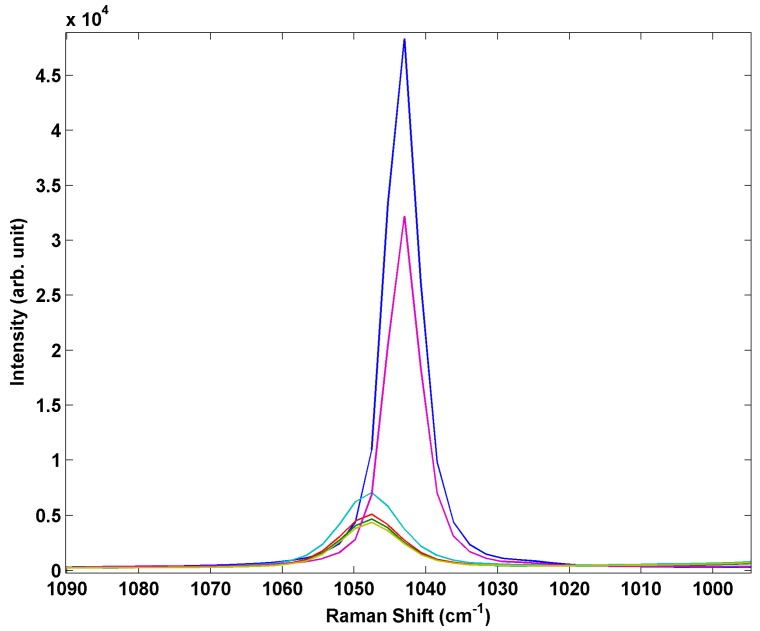
Comparison of Raman spectra of inkjet test materials containing both liquid and solid AN deposits.

In addition to forming various polymorphic crystal phases, ammonium nitrate is prone to deliquescence and can readily absorb water from the air in moderate relative humidity conditions potentially leading to the formation of supersaturated liquid AN drops [33]. Thus, in a real world detection scenario a sensor may be required to detect AN in the liquid form in humid environments. Conversion of the solid inkjet-printed particle arrays to liquid drops can easily be achieved even in a controlled humidity laboratory environment by the simple act of breathing on the arrays. This creates mixed arrays containing both solid crystalline particles and liquid particles. The liquid droplets are readily differentiated in the optical microscope by their appearance and the optical lensing behavior of the liquid. Figure 7 shows a series of Raman spectra from printed AN array with mixed solid and liquid AN deposits. The hydrated drops show an increase in intensity of the silicon band, a decrease in intensity of the NO_3_^−^ peak along with heterogeneous broadening and blue shifting as shown in Figure 8. This spectral variation is not particularly pronounced but should be measurable in a spectrometer with conventional spectral resolution and may suggest the printed arrays may be useful for distinguishing between crystalline and aqueous phase ammonium nitrate.

**Figure 9:**
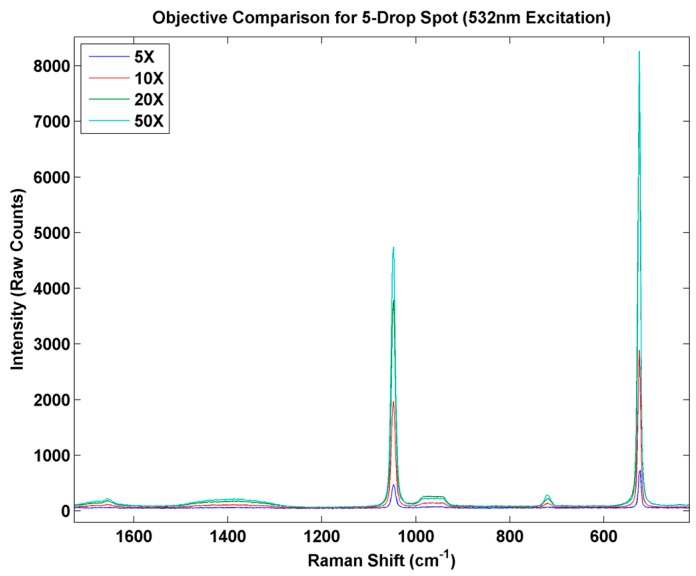
Micro Raman spectrum of individual AN particle prepared by inkjet printing showing the effect of microscope objective on response.

With each array element serving as a reproducible and single particle test sample, the influence of various experimental variables on the Raman response can be evaluated. For example, Figure 9 shows the response of the Raman microprobe to variation in the objective lens settings for one of the crystalline AN samples. Such uniformly-prepared samples can provide reproducible samples for optimization of the sensor setup prior to field studies.

**Figure 10:**
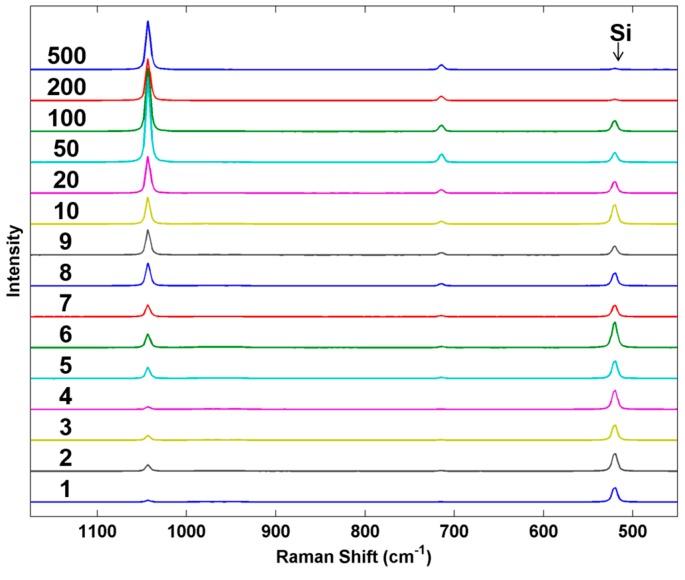
Average Raman spectra from 30 scans taken near the center of each particle from the AN series. Numbers on the left indicate the drops per spot used to create each analyzed particle. For smaller spot sizes (≤10 drops), spectra were collected over the entire particle.

As a final example, we examined the response of the Raman system to particles of increasing mass. Figure 10 shows a series of Raman spectra of individual AN particles prepared by using an increasing number of inkjet microdrops/spot printed from the standard AN solution. The average mass of each inkjet droplet was 44.184 ng or 44.184 pL ± 0.206 (*n* = 13, 0.46% RSD). Particle mass in the linear array of particles varied from 1.015 ng (1 drop/spot) to 515.11 ng (500 drops/spot). The spectra were collected using a 5× objective and show the average intensity of the AN Raman bands reaching a maximum between 20 and 50 microdrops per particle and then fluctuating beyond this. The Si band from the underlying substrate, however, continues to decrease with increasing particle mass. These trends will be highly dependent on the illumination and collection optics employed but demonstrates how particles with well controlled parameters could be used to characterize and potentially optimize a detection system. For this particular example, particle dimensions were not explicitly measured by ESEM on each of the particles to prevent possible electron beam damage prior to the Raman measurements. We anticipate in future work that the size calibration data shown in Figure 4 would be obtained from a separate calibration sample prepared under identical conditions as the sample to be analyzed. For calibration of sensors, the control over the fabrication process allows us to make individual particle deposits that could be used to evaluate a new technique for its sensitivity to particles of different masses (and sizes) in comparison to known actual threats. Such uniformly varying particles can be used to explore the fundamental limitations of a new detection sensor at an early stage of technology development.

## 5. Conclusions

A method has been developed to prepare trace detection test materials for spatially-resolved analytical methods. The procedures allow for the production of particle test materials with each particle having a well-defined concentration, particle size, shape, and position. These test materials can be used for evaluation of detector performance using samples that mimic (in terms of size and composition) real world contraband particles. The ability to precisely control the chemical and physical parameters of these materials makes them especially relevant for calibration and characterization of the performance of a sensor at an early stage of technology development when the fundamental limitations of a technique are still being evaluated. One example would be detection limit as a function of particle size. The high repeatability of the test materials also makes them potential candidates for certification studies where a high degree of confidence in the test materials is required.

**Figure 11:**
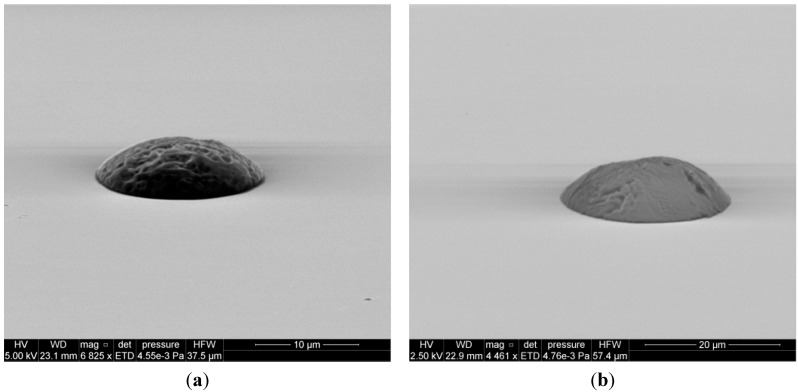

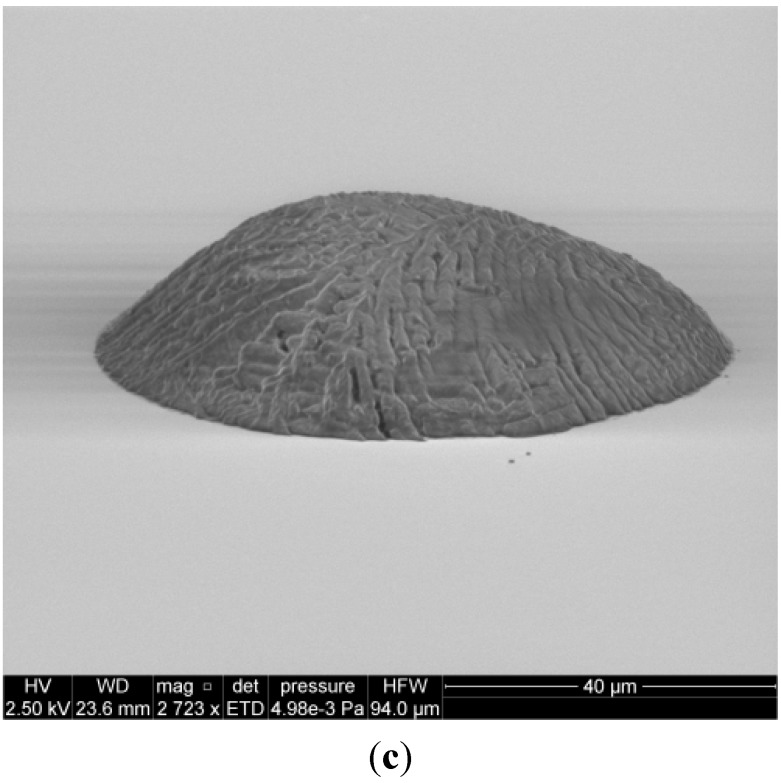
SEM images of RDX particles printed on the treated silicon surface. (note images are not at the same spatial scale). (**a**)1 Drop, ≈12 μm wide, 3 μm high; (**b**) 10 Drops, ≈27 μm wide, 7 μm high; (**c**) 100 Drops, ≈55 μm wide, 17.8 μm high.

While the focus of the current work has been on aqueous-based printing, many of the explosive threats for which standards are needed are not water soluble. Fortunately, the outlook for extension of this approach to a wide range of solvents is promising. The FTDS-coated silicon surface is partially oleophobic and gives contact angles of ≈45° for isopropanol and ≈73° for acetonitrile [34]. This has allowed us to also fabricate discrete particle arrays for analytes printed from these non-aqueous solvents. Figure 11 shows individual particles printed onto the silane treated silicon using a commercial acetonitrile standard solution containing 1 mg/mL of Cyclotrimethylenetrinitramine (RDX) with an individual inkjet drop volume of 20.793 ± 0.22 pL, RSD = 1.07%). Similar results have been obtained for trinitrotoluene (TNT), Pentaerythritol tetranitrate (PETN), and cyclotetramethylene-tetranitramine (HMX) printed from acetonitrile or acetonitrile water mixtures. In addition, the approach appears to be amenable to plastic-bonded explosives as we have been able to print Semtex 1A (PETN + polymer binder) dissolved in Tetrahydrofuran.

One area of concern for the practical use of these materials is the possibility of forming crystal polymorphs during the inkjet process. Rapid crystallization from the small volume of an inkjet drop may lead to the formation of polymorphs or metastable supersaturated liquid deposits. It has previously been demonstrated that inkjet-printed films of certain explosives, RDX for example, have a propensity to form metastable polymorphs when rapidly crystallized during printing. In the case of certain analytical techniques, such as Raman spectroscopy, these polymorphs may be spectrally distinct enough from bulk materials to result in misidentification of real threats [35]. The ramifications and mitigation of polymorph and metastable liquid phase formation during inkjet printing is currently an active area of investigation by several research groups. Finally, the chemical and physical stability of the inkjet materials needs to be evaluated to determine an acceptable lifetime for use after preparation. These studies are currently underway.
